# Effect of ammonia-free silver fluoride and silver diammine fluoride on immediate microshear bond strength of resin-modified glass ionomer to demineralised primary tooth dentine

**DOI:** 10.1007/s40368-026-01242-7

**Published:** 2026-06-19

**Authors:** A. Abedinzade Zare, M. Vafadar, M. Behroozibakhsh, A. Hashemian, M. Lari, S. Abtahi

**Affiliations:** 1 Department of Paediatric Dentistry, School of Dentistry, Tehran University of Medical Sciences, Tehran, Iran, Islamic Republic of; 2 Department of Dental Biomaterials, School of Dentistry, Tehran University of Medical Sciences, Tehran, Iran, Islamic Republic of; 3 Dental Research Center, Dentistry Research Institute, Tehran University of Medical Sciences, Tehran, Iran, Islamic Republic of; 4 School of Computing, Macquarie University, Sydney, Australia; 5 School of Engineering, Macquarie University, Sydney, Australia

**Keywords:** Silver diammine fluoride, Glass ionomer cements, Primary dentition, Bond strength, Dental caries

## Abstract

**Purpose:**

This study aimed to compare the effect of water-based ammonia-free silver fluoride (SF) and silver diammine fluoride (SDF) on the microshear bond strength (MSBS) of resin-modified glass ionomer (RMGIC) to artificially demineralised primary tooth dentine.

**Methods:**

Twenty-four extracted primary molars were prepared to expose mid-coronal dentine; two bonding sites per tooth (total = 48 specimens) were chemically demineralised (pH 4.8) to simulate active caries. Specimens were randomised into three groups (*n* = 16): SF (Riva Star Aqua), SDF (Cariestop 30%), and Control (distilled water). RMGIC (Riva Light Cure HV) was bonded and stored for 24 h at 37 °C. MSBS was measured using a universal testing machine, and failure modes were analysed using a stereomicroscope. Data were analysed using one-way ANOVA with Tamhane's T2 post hoc test (α = 0.05).

**Results:**

The SF group exhibited significantly lower bond strength (3.65 ± 1.80 MPa) than the Control (6.86 ± 3.15 MPa, *p* < 0.01) and SDF groups (6.65 ± 4.80 MPa, *p* < 0.05). No significant difference was found between SDF and Control (*p* > 0.05); however, the SDF group demonstrated greater variability, consistent with Weibull analysis. Adhesive failure predominated across all groups, with SDF showing more mixed failures.

**Conclusion:**

Water-based SF significantly compromised RMGIC adhesion to demineralised primary tooth dentine. SDF maintained comparable mean bond strength to the control but with higher variability. Ammonia-free SF formulations may reduce immediate RMGIC retention when used before restoration, and this interaction may warrant protocol optimisation in SMART-based applications.

## Introduction

Early childhood caries (ECC) remains a persistent global health burden, ranking among the most prevalent chronic diseases in children worldwide (Zou et al. 2022; Yang et al. 2025). Despite advancements in preventive strategies, untreated ECC frequently progresses to pulpal infection and odontogenic complications, necessitating invasive interventions that can compromise a child's oral health-related quality of life (Grund et al. 2015; Zaror et al. 2022). Consequently, paediatric dentistry has shifted toward minimally invasive approaches, prioritising tissue preservation and caries arrest without extensive cavity preparation (Gao et al. 2022; Garrocho-Rangel et al. 2023).

Silver diammine fluoride (SDF) has emerged as a cornerstone of this paradigm due to its dual mechanism of action: bactericidal silver ions and remineralising fluoride ions (Zhao et al. 2018; Seifo et al. 2019). Clinical trials and systematic reviews have consistently demonstrated high caries lesion arrest rates following SDF application in the primary dentition (Seifo et al. 2019). However, the clinical adoption of SDF is limited by significant aesthetic and practical drawbacks, primarily the permanent black staining of carious lesions (Seifo et al. 2020; Almuqrin et al. 2023; Burgess and Vaghela 2018). Furthermore, traditional formulations utilise ammonia as a stabiliser, which contributes to an unpleasant odour and has been associated with transient mucosal irritation and patient discomfort (Seifo et al. 2021). These limitations may negatively influence parental acceptance and treatment uptake, particularly for anterior primary teeth (Seifo et al. 2021).

To address these challenges, innovative water-based, ammonia-free silver fluoride (SF) formulations, such as Riva Star Aqua (SDI Ltd., Australia), have been developed. In vitro studies suggest that ammonia-free systems may exhibit improved cytocompatibility and reduced soft tissue irritation compared with conventional SDF formulations (Kim et al. 2022; Mulder et al. 2023). These products do not include ammonia and utilise a potassium iodide (KI) application step to precipitate silver iodide, thereby mitigating discolouration. The elimination of ammonia removes the source of pungent odour and potential mucosal irritation associated with conventional SDF. The two-step KI protocol also provides a visible application endpoint, as the creamy-white silver iodide precipitate transitions to clear precipitate (Mulder et al. 2024).

Nevertheless, alterations in chemical composition, ion release behaviour, and dentine surface interactions may influence subsequent adhesive procedures, particularly when restorative materials are placed immediately after silver fluoride application (Intajak et al. 2024).

In the Silver Modified Atraumatic Restorative Treatment (SMART) technique, silver fluoride-treated teeth are restored with resin-modified glass ionomer cement (RMGIC) (Natarajan 2022). The long-term success of SMART restorations depends heavily on adequate adhesion between RMGIC and the underlying dentine, as insufficient bonding can lead to restoration debonding, marginal leakage, secondary caries, and early failure (Fröhlich et al. 2022). While the effect of SDF on the bond strength of RMGIC has been extensively studied, results remain conflicting, ranging from no adverse effect to significant reductions in adhesion depending on application protocol and substrate condition (Intajak et al. 2024; Jiang et al. 2020; Lutgen et al. 2018). Importantly, most available studies focus on ammonia-containing SDF formulations and often involve sound or permanent tooth dentine, limiting their applicability to newer ammonia-free systems and primary teeth (Alessandro et al. 2024).

Microshear bond strength (MSBS) testing is particularly suited to the limited substrate area and structural fragility of primary teeth. Unlike micro-tensile testing, which requires mechanical sectioning and trimming that can induce micro-cracks and premature cohesive failures in primary tooth enamel and dentine, MSBS avoids these preparation-related artefacts (Assunção et al. 2020; Kharouba et al. 2023). Furthermore, MSBS offers a more uniform stress distribution than conventional macro-shear protocols and allows for standardised, regional measurements that better reflect the actual interfacial bond strength (Kharouba et al. 2023; Assunção et al. 2020).

It is currently unknown whether water-based, ammonia-free silver fluoride formulations adversely affect the adhesion of RMGIC to caries-affected primary tooth dentine. This represents a critical knowledge gap given the increasing clinical adoption of ammonia-free silver fluoride systems within minimally invasive paediatric restorative protocols. Therefore, this study aimed to evaluate the immediate MSBS of RMGIC to artificially demineralised primary tooth dentine following pretreatment with the commercially available ammonia-free SF/KI system (Riva Star Aqua) compared with a conventional SDF formulation (Cariestop 30%) and an untreated control, as applied according to their respective manufacturer protocols. The null hypothesis was that there would be no significant difference in MSBS among the three groups.

## Methods

### Study design and ethical considerations

**Fig. 1:**
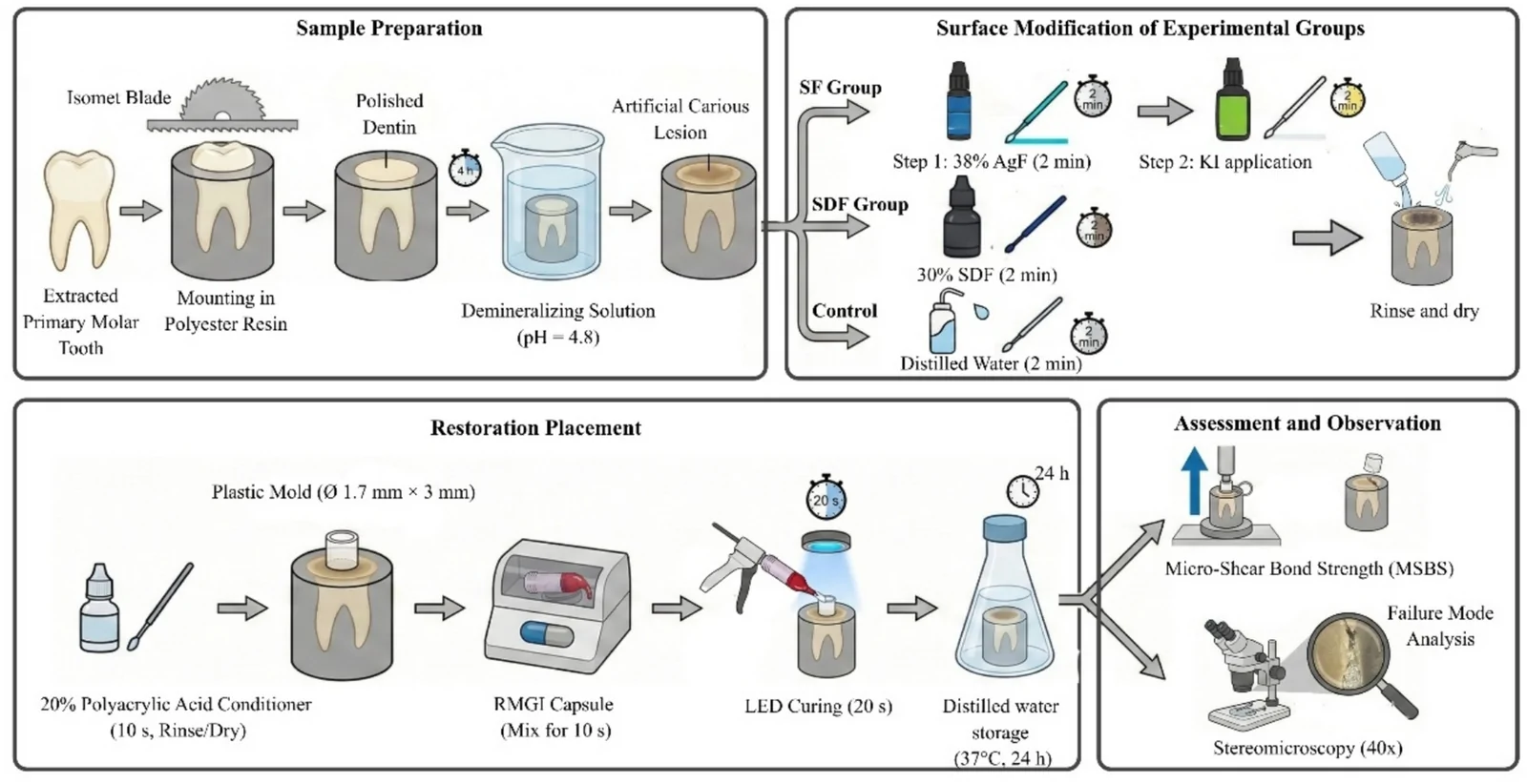
Schematic illustration of the procedures conducted in the current study. The illustration depicts a single bonding site for clarity and to avoid procedural confusion. In practice, two independent bonding sites were utilised per tooth block

This in vitro experimental study was approved by the Ethics Committee of Tehran University of Medical Sciences (IR.TUMS.BLC.1402.129) and is summarised in Fig. 1. The sample size was determined using G*Power software (v.3.1.9.2) based on data from a pilot study. A total of 48 specimens (16 per group) derived from 24 extracted primary molars (two specimens per tooth) were required to detect a significant difference with 80% power and an alpha error of 0.05. The specific materials, compositions, and manufacturers used in this study are detailed in Table 1.

**Table 1:** Materials used in the study

Brand name	Material type	Main composition*	Manufacturer
Riva star aqua	SF	Step 1: Silver fluoride (38%), water Step 2: Potassium iodide, water, and additives	SDI, Bayswater, VIC, Australia
Cariestop 30%	SDF	Silver diammine fluoride (30%), water, ammonia	Biodinâmica, Ibiporã, PR, Brazil
GC Cavity conditioner	Dentine Conditioner	20% Polyacrylic acid, 3% Aluminium chloride, water	GC Corp., Tokyo, Japan
Riva light cure HV (Capsules)	Resin-modified Glass Ionomer	Powder: Fluoroaluminosilicate glass Liquid: Polyacrylic acid, tartaric acid, HEMA, dimethacrylate monomers Mixing: Triturated (10 s)	SDI, Bayswater, VIC, Australia
Bluephase N	Polywave LED Curing Unit	Output irradiance: 1,200 mW/cm^2^ (Wavelength range: 385 – 515 nm)	Ivoclar Vivadent, Schaan, Liechtenstein

^*^Compositions based on manufacturer safety data sheets (SDS)

### Specimen preparation

Twenty-four extracted sound primary molars were disinfected in 0.5% chloramine-T for one week and stored in distilled water and tested within one month of extraction. Each tooth was embedded in self-curing polyester resin in cylindrical moulds such that the occlusal plane was parallel to the mould base. The enamel was sectioned using a precision saw (Mecatome T201A, PRESI, Eybens, France) under continuous water cooling to expose flat mid-coronal dentine. Surfaces were standardised by polishing with 400-, 800-, and 1000-grit silicon carbide papers for smear layer formation. Two non-overlapping bonding areas (each 1.7 mm diameter) were demarcated on the coronal dentine surface of each tooth block, with a minimum centre-to-centre separation of 3 mm to prevent mechanical and chemical interference between test sites, yielding 48 specimens from 24 teeth (two specimens per tooth).

Artificial ‘caries-like’ lesions were induced by immersing the specimens in a demineralising solution (0.05 M acetate buffer containing 2.2 mM CaCl₂, 2.2 mM NaH₂PO₄, and 0.05 M acetic acid; pH 4.8) for 4 h at 37 °C. This protocol creates a surface consistent with active dentinal caries (Tiba et al. 2022).

### Surface treatments

The prepared specimens were assigned randomly to three experimental groups (*n* = 16), and treatments applied to each surface are defined in Table 2. Randomisation was performed using a computer-generated random sequence (1:1:1 allocation). Specimens were coded numerically by an independent operator, and group assignment was concealed until the interventions were applied. Both bonding sites on each tooth were allocated to different experimental groups wherever possible, to distribute the within-tooth correlation across groups rather than within them.

**Table 2:** Experimental groups and detailed application protocols

Group	Agent	Application protocol
SF	Riva star aqua	1. Apply Step 1 (silver fluoride) using the silver brush and leave for 2 min 2. Immediately apply step 2 (potassium iodide) using the green brush 3. Continue applying step 2 until the creamy white precipitate turns completely clear 4. Rinse thoroughly with distilled water and gently air-dry
SDF	Cariestop	1. Apply the solution using a microbrush and allow it to react for 2 min 2. Rinse thoroughly with distilled water and gently air-dry
Control	Distilled water	1. Apply for 2 min and gently air-dry

### Restorative procedure

To standardise the bonding area, a plastic mould (1.7 mm diameter and 3 mm height) was used on each prepared dentine surface. Prior to restoration, dentine surfaces were conditioned with 20% polyacrylic acid (GC Cavity Conditioner, GC Corporation, Japan) for 10 s, rinsed, and dried. RMGIC capsules (Riva Light Cure HV, SDI, Australia) were activated and mixed in an amalgamator for 10 s. Capsules were loaded into the applicator, and the material was injected into the mould and light-cured for 20 s using an LED curing unit (Bluephase N, Ivoclar Vivadent). After removing the mould, the specimens were stored in distilled water at 37 °C for 24 h for complete setting (Elsattar et al. 2023).

### MSBS testing

The specimens were mounted in a universal testing machine (Z010, Zwick, Germany) with a wire loop (0.2 mm diameter) placed at the dentine-cement interface (Elsattar et al. 2023). A shear load was applied at a crosshead speed of 0.5 mm/min until failure. Bond strength (MPa) was calculated by dividing the failure load (N) by the bonding area (A).

### SEM/EDX surface characterisation

To qualitatively illustrate the dentine surface after SF pretreatment, one representative SF-treated specimen was examined by field emission scanning electron microscopy (FESEM) coupled with energy-dispersive X-ray spectroscopy (EDX/EDS) using a Tescan Mira3 FESEM (Tescan, Czech Republic).This analysis was intended to provide surface information only and not a comparative quantitative assessment among groups.

### Failure mode analysis

Fractured surfaces were examined under a stereomicroscope (Olympus SZX9, Tokyo, Japan) at 40 × magnification. Failure modes were classified as adhesive (failure at the dentine-material interface), cohesive (failure within the restorative material or dentine), or mixed (Carrera et al. 2016).

### Statistical analysis

Data were analysed using SPSS software (version 25.0, IBM Corp., USA). The Shapiro–Wilk test was used to assess normality. Because Levene’s test indicated heterogeneity of variances (*p* < 0.05), one-way ANOVA followed by Tamhane’s T2 post hoc test was used for pairwise comparisons. The significance level was set at α = 0.05. To address the nested data structure (two specimens per tooth), the randomisation protocol allocated both specimens from each tooth to different experimental groups; consequently, within each group all 16 specimens derived from 16 separate teeth, ensuring within-group independence for the ANOVA.

## Results

The normality of the data distribution was confirmed using the Shapiro–Wilk test (*p* > 0.05). However, Levene’s test indicated significant heterogeneity of variances among the experimental groups (*p* < 0.05), necessitating the use of the robust Tamhane’s T2 post hoc test for pairwise comparisons. One-way ANOVA revealed a statistically significant difference in MSBS among the groups (*p* < 0.05).

**Fig. 2:**
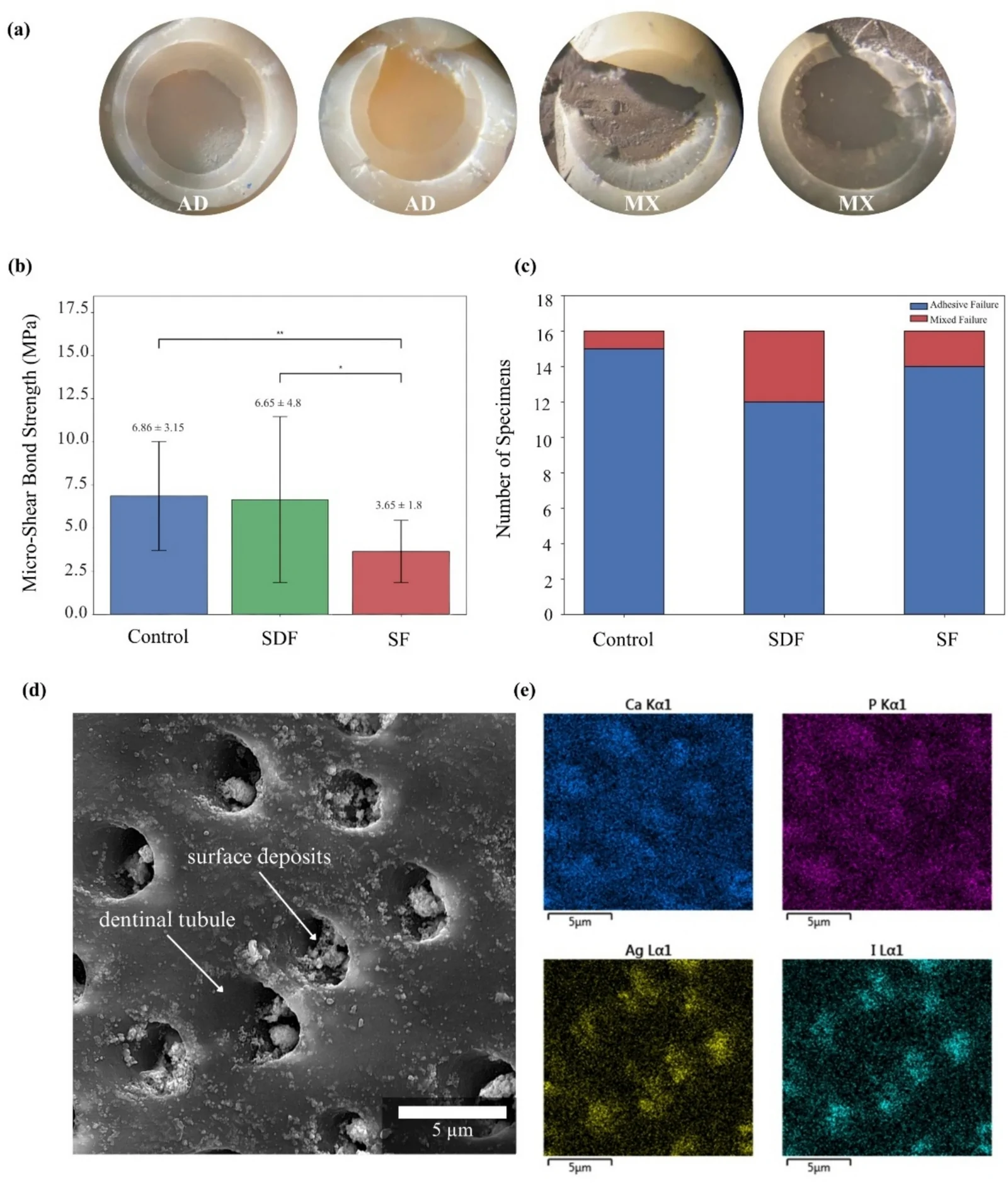
**a** Representative stereomicroscope images (40 ×) of failure modes after microshear bond strength (MSBS) testing: adhesive (AD) and mixed (MX). **b** Mean MSBS (MPa ± SD) for the Control, SDF, and SF groups; brackets indicate statistically significant pairwise differences (**p < 0.01; *p < 0.05). **c** Failure mode distribution per group (*n* = 16/group); predominantly adhesive failures were observed across all groups, with a higher proportion of mixed failures in the SDF group; cohesive failures were not observed. **d** Surface-view SEM micrograph of SF-treated demineralised primary tooth dentine showing exposed dentinal tubules and granular surface deposits. **e** Corresponding surface-view EDX elemental maps (Ca, P, Ag, and I); the co-localisation of Ag and I signals within the same areas as the particulate surface deposits is consistent with the formation of silver iodide (AgI) compounds on the dentine surface following SF/KI application

As illustrated in Fig. 2a-d, the Control group demonstrated the highest mean bond strength (6.86 ± 3.15 MPa), which was not statistically significantly different from the SDF group (6.65 ± 4.80 MPa; *p* > 0.05). In contrast, the water-based SF group exhibited the lowest mean bond strength (3.65 ± 1.80 MPa). This reduction was statistically significant compared to both the Control (*p* < 0.01) and the SDF group (*p* < 0.05). Notably, the SDF group displayed the largest standard deviation (4.80 MPa) and the widest range of values (1.27–18.27 MPa), indicating greater variability in bond strength values than the other groups.

SEM imaging of a representative SF-treated specimen showed exposed dentinal tubules with granular deposits on the surface and within/around tubule openings (Fig. 2d). Corresponding EDX mapping demonstrated localised Ag and I signal in the same region (Fig. 2e), suggesting the presence of residual silver/iodine-containing deposits after SF/KI application.

### Failure mode analysis

The distribution of failure modes is presented in Fig. 2c. Adhesive failure at the dentine-material interface was the predominant failure mode across all groups. The Control group exhibited 93.8% adhesive failures and 6.2% mixed failures, whilst the SF group showed 87.5% adhesive failures and 12.5% mixed failures. The SDF group showed a higher descriptive proportion of mixed failures (25.0%) and a lower proportion of adhesive failures (75.0%) than the other groups. No cohesive failures were observed within dentine or restorative material in any group.

### Weibull analysis

**Fig. 3:**
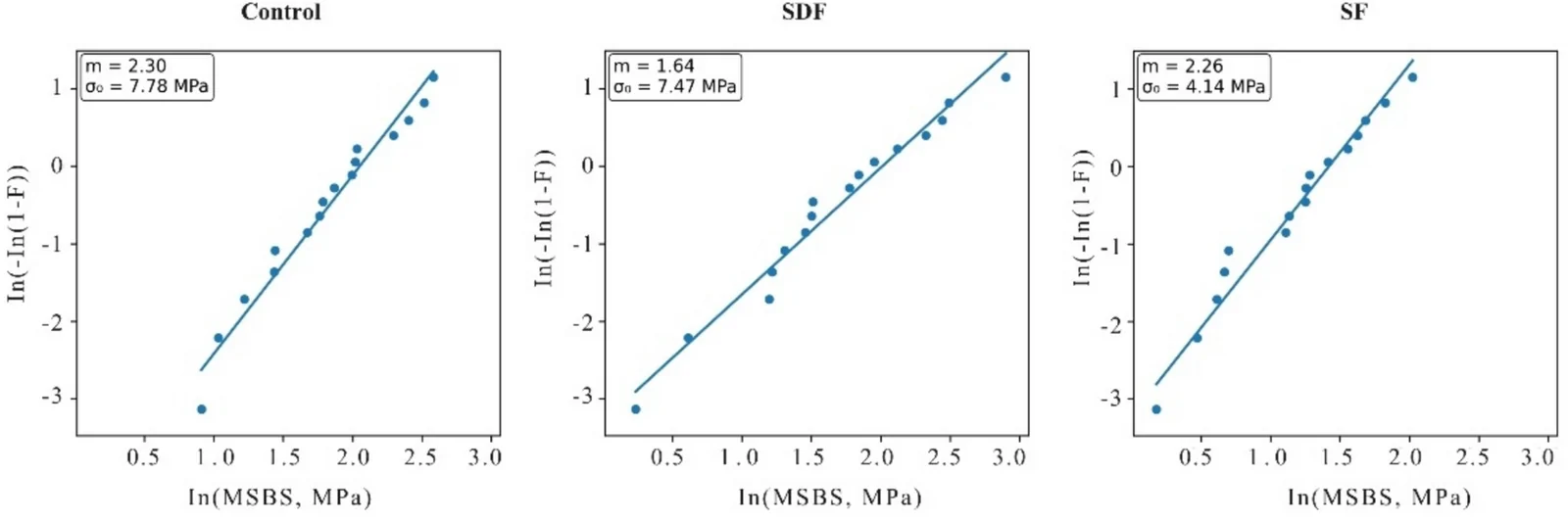
Weibull probability and two-parameter Weibull fits (lines; loc = 0) for MSBS of study groups. A higher Weibull modulus (m) indicates lower scatter (greater reliability), while σ₀ represents the characteristic strength (63.2% failure)

Weibull analysis revealed distinct reliability profiles among groups (Table 3). Overall, Control demonstrated the most consistent bonding performance (highest reliability and B10), whereas SDF showed greater scatter despite a similar overall strength level to Control. SF exhibited the weakest bond strength distribution and the lowest lower-bound reliability. Weibull probability plots showed good linearity across groups, supporting the suitability of the Weibull model for MSBS data (Fig. 3; Table 3).

**Table 3:** Weibull parameters of microshear bond strength (MSBS) for the three experimental groups

Group	Weibull modulus m (95% CI)	Characteristic strength σ₀ (MPa) (95% CI)	B10 (MPa)	
SF (Riva star aqua + KI)	2.26 (1.79–3.52)	4.14 (3.23–5.13)	1.53	
Control (Distilled water)	2.30 (1.86–3.44)	7.78 (5.99–9.45)	2.93	
SDF (Cariestop 30%)	1.64 (1.31–2.55)	7.47 (5.27–10.14)	1.89	

**Note:** The two-parameter Weibull distribution (location fixed at 0) was fitted by maximum likelihood estimation. The Weibull modulus (m) reflects data scatter (higher m = greater reliability). σ₀ denotes the characteristic strength corresponding to 63.2% failure probability. B10 is the stress at 10% probability of failure. Ninety-five percent confidence intervals were obtained by non-parametric bootstrap resampling (3000 iterations). MSBS (MPa) was calculated from debonding load (*N*) using the bonded area defined by a 1.7 mm diameter mould (area = 2.2698 mm^2^)

## Discussion

The present study investigated the adhesive interface between RMGIC and carious primary tooth dentine pre-treated with a novel water-based silver fluoride formulation compared to traditional silver diammine fluoride. The results necessitate the rejection of the null hypothesis, as the water-based SF formulation significantly compromised the bond integrity compared to both the control and the SDF.

Artificially demineralised primary tooth dentine was used to achieve substrate standardisation and to reduce the variability associated with naturally carious lesions. The demineralisation protocol was designed to reproduce key features of caries-affected dentine that are relevant to adhesion, including mineral loss and increased dentine porosity. This approach allows controlled comparison of the effects of different silver fluoride formulations on bond strength. However, artificially demineralised dentine does not fully replicate the biological and chemical complexity of naturally infected carious primary tooth dentine, such as lesion heterogeneity and bacterial byproducts (Abdelshafi et al. 2025). Therefore, the results should be interpreted as comparative rather than directly predictive of clinical performance.

MSBS testing was selected due to its suitability for primary tooth dentine and its ability to evaluate small, standardised bonding areas. This method facilitates specimen preparation and reduces the risk of premature cohesive failure during handling, which is a known limitation of micro-tensile testing when applied to primary teeth. However, MSBS testing is influenced by specimen geometry and non-uniform stress distribution at the loading interface, which may contribute to variability in measured bond strength values (Mourad 2018).

The fundamental finding of the present study was the significant reduction in bond strength in the water-based SF group (3.65 ± 1.80 MPa). Weibull analysis (Table 3) further highlighted distinct reliability profiles: although SDF and Control showed comparable characteristic strengths, the lower Weibull modulus for SDF indicates greater scatter and a higher probability of low-strength outcomes. In contrast, Control demonstrated the most predictable bonding performance (highest reliability and B10), whereas SF exhibited both reduced characteristic strength and a lower-bound reliability (B10), consistent with an overall shift toward weaker bonding rather than isolated outliers.

The significantly lower bond strength observed in the SF group relative to both the Control and SDF groups is likely related to differences in silver-ion stabilisation chemistry between the two formulations. Unlike SDF, in which ammonia stabilises silver in solution (Primus 2017), the ammonia-free SF system relies on a secondary application of saturated potassium iodide to precipitate free silver ions and reduce staining (See et al. 2024). This reaction produces silver iodide (AgI), an insoluble precipitate. Although the manufacturer recommends rinsing until the precipitate clears, the present findings suggest that residual silver/iodine-containing deposits may remain on or within the demineralised dentine surface after SF/KI application. Such deposits may interfere with intimate contact between RMGIC and the dentine substrate, thereby reducing effective micromechanical and ionic interaction at the interface.

This interpretation is partly informed by our qualitative SEM/EDX observations (Fig. 2d–e), which showed surface deposits with localised Ag and I signal, suggesting residual silver/iodine-containing precipitates after SF/KI application and washing. To contextualise these findings within the existing evidence base, Table 4 summarises key recent studies evaluating the adhesive interface of silver-treated dentine (Knight et al. 2006; Soliman et al. 2021; Ajchareeya et al. 2025; Puwanawiroj et al. 2018; Koizumi et al. 2016).

**Table 4:** Comparative summary of recent studies evaluating the effect of SDF and SF on the bond strength of restorative materials

Author	Substrate and sample size	Groups and intervention	Bond strength test	Restorative material	Outcome
Current study	Demineralised primary molar dentine (*n* = 16 per group)	G1: Control (distilled water) G2: SF + KI (Riva Star Aqua) G3: SDF (Cariestop 30%)	Microshear	RMGIC (Riva Light Cure HV, SDI)	*G1*: 6.86 MPa *G2*: 3.65 MPa (*p* < 0.01) *G3*: 6.65 MPa (NS) *Conclusion*: SF + KI significantly reduced adhesion
(Knight et al. 2006)	Permanent third molar dentine (*n* = 15 per group)	G1: AgF + KI (washed) G2: AgF + KI (air dried)	Shear	GIC (Fuji VII, GC)	*G1*: 2.83 MPa *G2*: 1.49 MPa (*p* < 0.05) *Conclusion*: Leaving the reaction precipitate significantly reduced the bond strength
(Soliman et al. 2021)	Sound primary molar dentine (*n* = 20 per group)	G1: Control (distilled water) G2: SDF (38%, rinsed)	Shear	RMGICC (Fuji II LC, GC)	*G1*: 4.40 MPa *G2*: 8.28 MPa (*p* < 0.0001) *Conclusion*: SDF significantly increased bond strength
(Ajchareeya et al. 2025)	Artificial carious human dentine (*n* = 16 per group)	G1: Control G2: SDF immediate G3: SDF + thermocycling	Micro tensile	Universal adhesive (Single Bond Universal, 3M)	*G1*: 24.28 ± 2.27 MPa *G2:* 14.20 ± 3.32 MPa (*p* < 0.001) *G3:* 18.26 ± 5.63 MPa. *Conclusion*: Immediate bonding reduced; durability improved after ageing
(Puwanawiroj et al. 2018)	Carious primary tooth dentine (*n* = 15 per group)	G1: Control (deionised water) G2: SDF (38%, rinsed)	Micro tensile	GIC (Fuji IX GP Extra, GC)	*G1*: 6.3 ± 4.6 MPa *G2*: 7.4 ± 5.1 MPa *Conclusion*: SDF did not adversely affect bonding
(Koizumi et al. 2016)	Sound bovine incisor dentine (*n* = 10 per group)	G1: Control (dentine conditioner) G2: SDF + KI (Riva Star)—rinsed	Shear	GIC (Fuji VII, GC)	*G1*: 4.8 MPa *G2*: 2.7 MPa (*p* < 0.05) *Conclusion*: KI precipitate is associated with reduced bonding

The present findings should be interpreted in the context of marked methodological differences across the literature, including dentine substrate type, silver formulation, use of potassium iodide, restorative material, and bond strength method. Studies evaluating SDF alone have often reported preserved bonding performance, whereas systems involving silver followed by potassium iodide have shown a greater potential to alter the adhesive interface.

Our finding that the SF/KI system reduced bond strength is more closely aligned with potassium-iodide-associated silver systems such as that reported by Koizumi et al., whereas studies evaluating SDF alone, including Puwanawiroj and colleagues, have generally reported preserved bonding performance (Puwanawiroj et al. 2018; Koizumi et al. 2016). Whilst some studies utilising sound dentine have reported maintained or improved bonding with silver treatments, the hyper-mineralised surface layer formed on sound dentine likely provides a stable base for adhesion that is absent in the demineralised lesions used in the present study (Cardenas et al. 2021; Sa’ada et al. 2021).

It is important to acknowledge a key interpretive constraint of this experimental design. The SF group received a two-step SF/KI protocol (Riva Star Aqua), whereas the SDF group (Cariestop 30%) did not include a KI step. The observed difference in bond strength between these groups therefore reflects the integrated effect of both the change in silver fluoride carrier chemistry (ammonia-containing versus ammonia-free) and the presence of the KI precipitation step. The independent contribution of potassium iodide to bond strength reduction is well supported in the literature: It was demonstrated that adding KI to SDF significantly reduced glass ionomer bond strength to dentine (Koizumi et al. 2016), and another study similarly showed that the AgI precipitate formed during KI application was strongly associated with reduced adhesion (Knight et al. 2006). The present findings are therefore most appropriately interpreted as reflecting the effect of the two-step SF/KI clinical protocol, rather than the effect of ammonia absence in isolation.

Beyond the physical obstruction of the AgI precipitate, the chemical environment created by the water-based formulation may further exacerbate adhesive failure. The water-based agent has been reported to contain a higher effective silver ion concentration compared to traditional 30% SDF, which Ko and colleagues (Ko et al. 2020) demonstrated can lead to heavier, more obstructive precipitation. Furthermore, the interplay between pH and enzymatic activity plays a critical role in bond durability. SDF creates a highly alkaline environment (pH ~ 9.4) that inhibits the activity of Matrix Metalloproteinases (MMPs), thereby preserving the collagen matrix. In contrast, the neutral pH (~ 7.4) and high-water content of the ammonia-free formulation may be more permissive to matrix metalloproteinase activity compared to the highly alkaline environment created by SDF. As suggested by Alessandro and colleagues, this neutral environment could permit enzymatic degradation of the collagen network at the interface, further weakening the hybrid layer compared to the collagen-stabilising alkaline environment of SDF (Alessandro et al. 2024).

Conversely, the SDF group (6.65 ± 4.80 MPa) exhibited a mean bond strength statistically comparable to the Control. This aligns with Soliman and colleagues, suggesting that the metallic silver and silver phosphate byproducts of SDF are sufficiently permeable to allow RMGIC interaction (Soliman et al. 2021). However, the high standard deviation observed in this group indicates significant technique sensitivity. The bond strength values ranged widely, suggesting that SDF application may result in variable interfacial silver deposition even after rinsing. A thin, well-infiltrated layer may reinforce dentine, while a thick, precipitate layer acts as a cohesive weak link at the adhesive interface. This unpredictability serves as a clinical caveat: while SDF is chemically compatible with RMGIC, the resulting bond consistency is inherently variable.

The analysis of failure modes revealed a distinct behavioural pattern in the SDF group, characterised by the highest prevalence of mixed failures (25%). This contrasts with the Control group, which, despite exhibiting the highest mean bond strength, failed predominantly at the adhesive interface (93.8%). This discrepancy suggests that SDF treatment fundamentally alters the fracture mechanics of the dentine surface. The deposition of silver-containing reaction products is believed to increase surface microhardness and structural heterogeneity of the substrate (Menzel et al. 2023; Sayed et al. 2020). Consequently, stress is not uniformly distributed along a single plane as it is in the Control group. Instead, the crack propagation in SDF-treated specimens is forced to deviate through the varying densities of the silver-impregnated zone, leading to a complex, mixed fracture pattern. This observation is consistent with the high standard deviation observed in this group, suggesting that the interface may function as a complex, variable zone of silver infiltration rather than a simple adhesive boundary.

The clinical implications of these findings are directly relevant to the SMART technique, in which silver fluoride pretreatment precedes immediate RMGIC placement in the same visit. When Riva Star Aqua (SF/KI) is used as the pretreatment agent, the subsequent RMGIC bond to demineralised primary tooth dentine may be significantly compromised relative to no pretreatment. Clinicians should ensure thorough rinsing until the AgI precipitate is completely cleared before applying the polyacrylic acid conditioner, as residual deposits may interfere with RMGIC adhesion. In contrast, when SDF is used as the pretreatment agent, mean bond strength was comparable to the untreated control, suggesting that SDF-based SMART protocols may be less susceptible to adhesive compromise, albeit with greater bonding variability. These findings should be considered when selecting and applying silver fluoride agents in SMART restorative protocols.

### Limitations and future directions

This study has several limitations. First, artificially demineralised primary tooth dentine does not fully replicate the biological complexity of naturally carious primary tooth dentine. Second, bond strength was assessed after 24 h of storage, reflecting immediate adhesion rather than long-term durability under ageing conditions. Third, SEM/EDX surface characterisation was performed on a single representative SF-treated specimen for qualitative illustration only; comparative characterisation of SDF-treated and control specimens was not conducted. Fourth, the SF group used a two-step SF/KI protocol (Riva Star Aqua) while the SDF group used a single-step formulation (Cariestop 30%) without KI, precluding isolation of the independent effects of ammonia and potassium iodide on bond strength. Future studies should employ a factorial design incorporating AgF alone, AgF + KI, SDF alone, and SDF + KI groups to delineate these contributions, evaluate clinically relevant ageing conditions, and characterise residual interfacial deposits in relation to bond durability. Further studies using naturally carious primary tooth dentine are needed before clinical recommendations can be made.

## Conclusions

Within the limitations of this in vitro study, ammonia-free SF/KI pretreatment reduced the immediate microshear bond strength of RMGIC to artificially demineralised primary tooth dentine, whereas SDF showed mean bond strength values comparable to the control but greater variability.

## Data Availability

The data presented in this study are available on request from the corresponding author.
